# Concentrated Growth Factors Combined with Lipopolysaccharide Stimulate the In Vitro Regenerative and Osteogenic Activities of Human Dental Pulp Stem Cells by Balancing Inflammation

**DOI:** 10.1155/2022/2316666

**Published:** 2022-12-15

**Authors:** Negin Firouzi, Hamid Reza Yavari, Saeed Rahimi, Leila Roshangar, Ramtin Chitsazha, Mohammad Amini

**Affiliations:** ^1^Department of Endodontics, Shiraz University of Medical Sciences, Shiraz, Iran; ^2^Department of Endodontics, Tabriz University of Medical Sciences, Tabriz, Iran; ^3^Department of Histology, Tabriz University of Medical Sciences, Tabriz, Iran; ^4^Department of Periodontics, Shiraz University of Medical Sciences, Shiraz, Iran; ^5^Immunology Research Center, Tabriz University of Medical Sciences, Tabriz, Iran

## Abstract

**Aim:**

We investigated the long-term effects of exposure to concentrated growth factors (CGFs) on the regenerative properties of dental pulp stem cells (DPSCs) in the presence and absence of lipopolysaccharide (LPS) as a proinflammatory agent.

**Methods:**

DPSCs were cultured with CGF at different concentrations of LPS (0.1, 1, and 10 *µ*g/ml) for 21 days. Then, using MTT and scratch assays, the cell viability and migration were examined. Osteogenic stimulation was evaluated by alkaline phosphatase (ALP) staining and Sirius Red staining, which determined the ALP activity and collagen levels, respectively. The expression levels of osteogenic markers were quantified using the qRT-PCR method. One-way analysis of variance (ANOVA) and Tukey's HSD test were used to analyze differences between groups.

**Results:**

Long-term treatment of DPSCs with CGFs reduced LPS-induced cell death. Moreover, CGF and LPS (1 *µ*g/ml), either in combination or alone, improved the DPSC migratory ability and caused a significant increase in osteogenic stimulation through the upregulation of collagen levels and ALP activity. Additionally, CGFs significantly upregulated RUNX2, DSPP, OCN, and OPN mRNA levels (as osteogenic markers), while LPS (1 *µ*g/ml) only significantly increased OCN overexpression.

**Conclusion:**

Our findings are evidence that CGF could be a promising agent to induce dentin-pulp complex healing in long-term chronic inflammation.

## 1. Introduction

Inflamed dental pulp and apical periodontitis are prevalent chronic inflammatory dental diseases predominantly caused by microbial infections [1, 2]. In response to microbial products, the pulp can stimulate immune responses, eventuating in pulp inflammation [3]. Despite its unfavorable impacts on dental pulp, low-degree inflammation is vital for dentin-pulp complex healing. Proinflammatory regulators stimulate dental pulp stem cell (DPSC) differentiation to odontoblast-like cells and lead to the mineralization of the tooth [4, 5]. However, severe inflammation prevents the regeneration of tissues, and this biphasic biological response to inflammation is known as hormesis [6]. Despite various treatment approaches (e.g., apexification and apical barrier techniques), chronic inflammation often remains in the pulp, inducing the constant loss of normal tissues and impairing innate repair abilities [7]. These issues indicate the importance of developing novel approaches to pulp regeneration, thereby reestablishing the pulp-dentin structure's normal function in maintaining dental pulp vitality.

Stem cells are considered a precious resource for developing and regenerating tissues due to their specific features, such as self-renewal, differentiation, and plasticity [8]. The adult stem cell's activity is affected by innate genetic programming and the stimulatory/inhibitory signals received from the environment. These cells postnatally regenerate unhealthy or injured tissues [9]. Notably, DPSCs represent a rich source of multipotent mesenchymal progenitor cells that help to replace lost odontoblasts [10]. Although these cells indicate high multipotency and proliferative capacity, drawbacks in the clinical application of these cells have been reported. Biomedical engineering innovations like scaffolds and biomaterials may improve these stem cells' cellular viability, differentiation, and therapeutic effectiveness in dental pulp regeneration [11].

Concentrated growth factors (CGFs) are the latest generation of platelet concentrate products with a dense fibrin matrix that comprises various growth factors, including platelet-derived growth factor-BB (PDGF-BB), transforming growth factor *β*-1 (TGF-*β*1), insulin-like growth factor-1 (IGF-1), vascular endothelial growth factor (VEGF), and basic fibroblast growth factor (bFGF). As a bioscaffold, CGFs participate in stem cells' attachment, proliferation, and differentiation and regulate the tissue regeneration process [12]. CGFs modulate numerous biological processes in stem cells, such as those in bone marrow stromal cells, mesenchymal stem cells, and periodontal ligament cells. In particular, treatment with CGF promoted the odontogenic and osteogenic potential of DPSCs in vitro, showing great significance in dental pulp regeneration [13]. Moreover, CGFs may exert desirable impacts on tissue regeneration, osteoclastogenesis, and bone formation during inflammation induced by lipopolysaccharides (LPS) by modulating the expression of proinflammatory cytokines such as interleukin-6 (IL-6), IL-1*β*, and TNF-*α* in DPSCs [14].

A necrotic pulp is caused by microorganisms, such as Gram-negative bacteria [15]. LPS, as the primary molecules in the cellular wall of these bacteria, are utilized to model inflammation by being the primary toxic factor involved in bacteria-induced immune responses [16]. As mentioned, controlling this situation with bioscaffolds is essential to induce pulp regeneration. CGFs reportedly optimize the balance between the inflammatory and regenerative responses in the infected pulp and influence the proliferation, differentiation, and migration of LPS-stimulated pulp stem cells [14]. However, the long-term therapeutic effects of CGFs on the mineralization of DPSCs are yet to be investigated. With the aim of clinical usage, it is essential to explore the efficiency of CGF in situations similar to the clinical conditions of immature necrotic teeth as a consequence of inflammation induced by metabolic products of bacteria. Therefore, this in vitro study evaluated the long-term effects of CGF on the proliferation, migration, and differentiation of cultured DPSCs under LPS-induced inflammation.

## 2. Materials and Methods

### 2.1. Preparation of CGF

This experimental study was approved by the Ethics Committee of Tabriz University of Medical Sciences with code IR.TBZMED.VCR.REC.1399.392. After obtaining informed consent from each candidate, fresh venous blood samples (10 ml) were voluntarily donated by healthy adults (*n* = 5). Blood samples were placed in sterile tubes without anticoagulant solutions and immediately centrifuged according to the following process: 30 s acceleration, 2 min at 692 g, 4 min at 547 g, 4 min at 692 g, 3 min at 855 g, and 36 s deceleration (MEDIFUGE™, Silfradentsrl, S. Sofia, Italy). After centrifugation, four layers (from top: serum, fibrin buffy coat, CGF, and red blood cells (RBCs)) were observed in the tube. The CGF clots were then removed from the tube with tweezers and separated from surrounding phases using sterile microscopic scissors. Subsequently, the clots were blended and set on a sterile gauze to discard excess serum, and then, CGF clots were minced, homogenized, and placed at −80°C for 1 h; after that, they were centrifuged at 3000 rpm for 10 min at room temperature. The superior liquid (CGFs) was sifted using a 0.22-mm sterile syringe filter (Memberan, CA) and put away at −80°C till use. [17].

### 2.2. Cell Culture

Dental pulp stem cells (DPSCs) were obtained from the Iranian Biological Resource Center (Tehran, Iran). The cells were cultured in low-glucose DMEM (Dulbecco's Modified Eagle Medium) containing 10% fetal bovine serum (FBS) and 1% penicillin-streptomycin. The cells were maintained in an incubator at 37°C and 5% CO_2_. All experiments were performed after 3–5 consecutive cellular passages. DPSCs were divided into the six groups as mentioned in Table 1.

### 2.3. Sample Preparation for SEM Analysis

**Figure 1:**
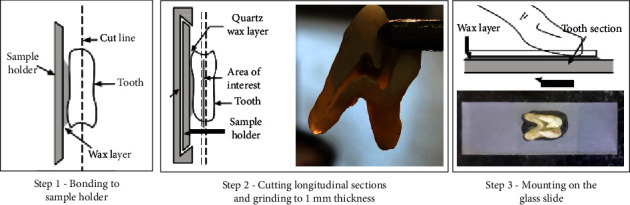
Preparation of tooth sections for SEM analysis.

Impacted third molars were collected for SEM analysis. Teeth were individually inserted and stored in 10% formaldehyde plastic vials until use. Samples were sectioned longitudinally under water cooling with a diamond saw rotating at 500 rpm (Isomet, Buehler Ltd., Lake Bluff, IL, USA). Each tooth was sliced to 1 mm thickness using a linear precision cutting machine (Figure 1). After mounting each tooth slice on a glass slide, samples were immersed in 5.25% NaOCl for 24 h for sterilization. Specimens were rinsed and soaked in 1 × phosphate-buffered saline (PBS) for one week to remove residual agents and stored in a serum-free medium. Then, DPSCs at a density of 1 × 10^3^ cells/well were seeded on each disc. The cells were treated for 21 days and then fixed in 2.5% glutaraldehyde solution (Sigma-Aldrich, USA) at 4°C for 1 hour before being dehydrated by gradient ethanol. Treated DPSCs were observed using scanning electron microscopy (SEM; TESCAN MIRA3 FEG).

### 2.4. ELISA Assay

An ELISA assay was used to detect and quantify protein levels of TNF-*α* in the treatment groups. A specific antibody for TNF-*α* was precoated onto the microwells. After incubation, proteins were captured by the coated antibody and samples were washed extensively. To detect the captured proteins, another antibody specific to proteins was added. Then, a horseradish peroxidase (HRP)-conjugated antibody was added to the developing signal, followed by the tetramethylbenzidine (TMB) reagent. The solution containing sulfuric acid was added to samples to stop color development, and the color intensity indicating the bound protein quantity was measured at 450 nm.

### 2.5. Cell Proliferation Assay

Cell viability was assessed using the MTT assay. DPSCs at a density of 2 × 10^3^ cells/well were incubated for 24 hr in 96-well plates containing DMEM and 10% FBS. The cells were exposed to CGF, LPS (0.1, 1, or 10 *µ*g/ml), or a combination of 1 *μ*g/mL LPS, and 1 × CGF. During the 21-day study period, the culture media were refreshed every two days. The cells without any treatment were considered the control group. After incubation, MTT solution (2 mg MTT powder in 1 ml PBS) was added to the wells and plates were maintained in the incubator for 4 hr. Then, the solution was removed. To dissolve the formazan crystals, 0.1 ml dimethyl sulfoxide (DMSO) was added to each well and the plates were shaken at 100 rpm for 10 minutes in the dark. The optical density (OD) was measured using a microplate reader (Bio-Rad Laboratories, Hercules, CA, USA) at a scale of 450 nm. After comparing the results of the different groups, a concentration of 1 × CGF was selected for further experiments.

### 2.6. Wound Healing Assay

A wound-healing assay was used to evaluate cellular mobility through treatment with CGFs in the presence of LPS. The cells were seeded into 24-well plates at a density of 1 × 10^5^ cells/well and cultivated for 24 h. Then, the cells were treated for 21 days with the previously mentioned treatments in 6 groups. The culture media were replaced every two days. The cells without any treatment were considered the control group. On day 21, an artificial gap was created across the cell monolayers using yellow pipette tips and maintained for 12 h. Images of cell migration into the wound area were captured at 0 h and 12 h after wound creation using an inverted microscope (Optika, Bergamo, Italy). Wound healing was measured by the area method in the following formula:

(1) $$\begin{array}{c} Migration\;area\;percentage\;at\;12h=\frac{Area\left(0h\right)-Area\left(12h\right)}{Area\left(0\right)}\times 100\%\text{.} \end{array}$$

### 2.7. Detection of Alkaline Phosphatase Activity

Alkaline phosphatase (ALP) is the most commonly suggested biochemical marker for osteoblast activity. DPSCs were exposed to three different concentrations of LPS and/or CGF in 6 groups for 21 days. The osteocytes differentiation medium (Bonbiotech, Iran) with different treatments was changed every two days. For ALP staining, after removing the medium from the wells, the cells were fixed in 70% ethanol for one hour. Then, the cells were washed with deionized water three times and a solution of 5-bromo-4-chloro-3-indolyl tetrazolium phosphate (Beyotime, Shanghai, China) was added to each well. The cells were washed several times and then photographed. Also, for quantitative exploration, 10% cetylpyridinium chloride (Sigma-Aldrich) was used to extract the amount of dye. The absorbance was measured at 562 nm on a microplate reader (BioTek, Winooski, USA).

### 2.8. Detection of Collagen (Sirius Red Staining)

Sirius Red staining was used to evaluate the amount of collagen in the discs after cultivation with CGF and/or LPS in 6 groups for 21 days. The wells were stained in picro-Sirius Red for one hour. Then, the cells were washed in two changes of acidified water. Vigorous shaking led to water removal from the slides before the samples were dehydrated in three changes of 100% ethanol. The samples were cleaned in xylene and placed in a resinous medium. Finally, after many steps of washing, the samples were photographed.

### 2.9. Quantitative Real-Time PCR Analysis (qRT-PCR)

Cellular RNA was obtained from treatment groups using the RiboEX reagent (GeneAll Biotechnology, Seoul, South Korea) as per the manufacturer's instructions. In this test, the cells without any treatment and those treated by osteogenic medium (OM) were considered negative and positive controls, respectively. The concentration and quality of RNAs were measured using the NanoDrop2000 spectrophotometer (Thermo Scientific, Waltham, MA, USA). After RNA extraction, reverse transcription was performed via the BIOFACT (Korea) kit to assess the mRNA expression levels of target genes according to protocols supplied by manufacturers. To determine the mRNA expression levels of dentin matrix protein 1 (DMP-1), dentin sialophosphoprotein (DSPP), osteopontin (OPN), runt-related transcription factor 2 (Runx2), and osteocalcin (OCN), real-time PCR was performed using the SYBR Premix Ex Taq (Takara Bio, Otsu, Shiga, Japan) under the following cycling conditions in a LightCycler® system (Roche Diagnostics, Mannheim, Germany): initial denaturation at 95°C for 10 min, 45 cycles of 95°C for 10 s, and 1 min at 60°C. The reaction mixture had a total volume of 20 *μ*L, including 8 *μ*L of ddH_2_O, 1 *μ*L of cDNA (1000 ng), 1 *μ*L of the forward and reverse primers (4 pmol), and 10 *μ*L of SYBR® Green Realtime PCR Master Mix. The relative gene expression was calculated via the comparative 2^−∆∆CT^ method. The mRNA expression of the target genes was normalized to GAPDH as the endogenous reference gene. The primer sequences are summarized in Table 2.

### 2.10. Statistical Analysis

Differences between groups were detected using analysis of variance (ANOVA), and a *p* value below 0.01 indicated a statistical significance. Individual differences between groups were evaluated using the Tukey's honest significant difference (HSD) test (*p* < 0.01). All values are reported as the mean ± standard deviation (SD) of triplicated experiments. GraphPad Prism 6 (GraphPad, San Diego, CA) and ImageJ were used for statistical analysis.

## 3. Results

### 3.1. Concentrated Growth Factor Demonstrated Excellent Cytocompatibility

**Figure 2:**
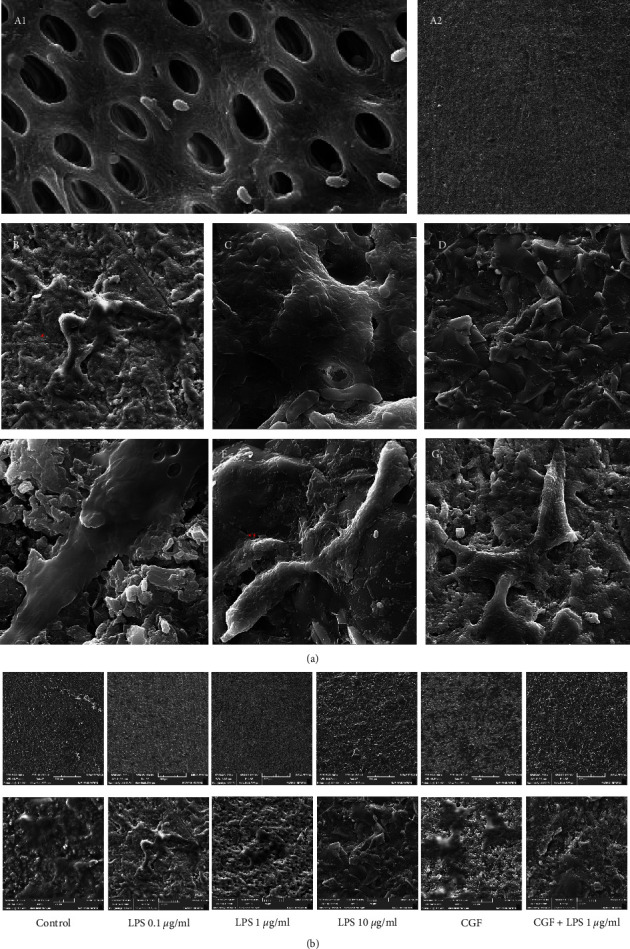
(a) Scanning electron microscopy (SEM) of dental pulp stem cells (DPSCs) cultured onto dentin discs (A1, 2) treated with LPS 0.1 *µ*g/ml (B), LPS 1 *µ*g/ml (C), LPS 10 *µ*g/ml (D), CGF (E), LPS plus CGF (F), and the control group lacking LPS/CGF (G). The dentin surface and dentinal tubules can be seen in part (A) Adhesion of DPSCs (^*∗*^) is evident with clear cytoplasmic extensions (^*∗∗*^) in all groups except for the LPS 10 *µ*g/ml group. (b) Scanning electron microscopy (SEM) of dental pulp stem cells (DPSCs) cultured onto treated dentin discs. Under 200x magnification (upper row), it is evident that the highest cell number was in the concentrated growth factor (CGF) group, followed by the lipopolysaccharide (LPS) 0.1 *µ*g/ml and control groups. There are nearly no viable cells in the LPS 10 *µ*g/ml group. There was probably higher cell proliferation and attachment in the CGF + LPS 1 *µ*g/ml group compared with LPS 1 *µ*g/ml.

The morphology and adhesion of DPSCs on dentin discs after treatment with LPS and/or CGF after 21 days are shown in Figures 2(a), 2(b). SEM analysis revealed better spreading and growth of DPSCs in the LPS 0.1 *μ*g/mL, LPS 1 *μ*g/mL, CGF, and LPS + CGF groups. However, there were limited recognizable DPSCs in the LPS 10 *μ*g/mL group, suggesting the cytotoxic properties of higher concentrations of LPS. By adding CGF to 1 *μ*g/mL of LPS, cell adhesion was promoted and cytoplasmic extensions appeared.

### 3.2. The Effect of Concentrated Growth Factor and Lipopolysaccharide on TNF-*α* Levels in Dental Pulp Stem Cells

**Figure 3:**
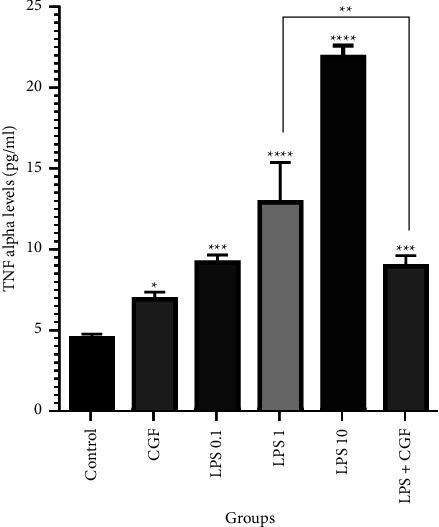
TNF-*α* levels at the end of the study in the treatment groups; concentrated growth factor (CGF) significantly lowered TNF-*α* production by lipopolysaccharide (LPS) in dental pulp stem cells; ^*∗∗∗∗*^*p* < 0.0001, ^*∗∗∗*^*p* < 0.001, ^*∗∗*^*p* < 0.01, and ^*∗*^*p* < 0.05.

Inflammation was assessed in each group before and after the 21-day study period according to the TNF-*α* level. In the control group, treatment of DPSCs with CGF significantly (*p* < 0.05) increased the TNF-*α* level from 4.47 ± 0.25 to 6.90 ± 0.42 pg/ml. Also, as expected, treating cells with LPS led to an increase in TNF-*α* levels in a dose-dependent manner; exposure of DPSCs to LPS 0.1 *µ*g/ml, LPS 1 *µ*g/ml, and LPS 10 *µ*g/ml increased the levels of TNF-*α* to 9.17 ± 0.45 (*p* < 0.001), 12.90 ± 2.64 (*p* < 0.0001) and 21.90 ± 0.70 (*p* < 0.0001) pg/ml, respectively. However, the combination of CGF and LPS 1 *µ*g/ml was able to promote TNF-*α* production to 8.93 ± 0.64 pg/ml, which was significantly (*p* < 0.01) lower than LPS 1 *µ*g/ml alone (Figure 3). These results suggest that CGF may stabilize LPS-induced TNF-*α* upregulation in favor of inducing chronic inflammation in long-term conditions.

### 3.3. The Effect of Concentrated Growth Factor and Lipopolysaccharide on the Viability of Dental Pulp Stem Cells

**Figure 4:**
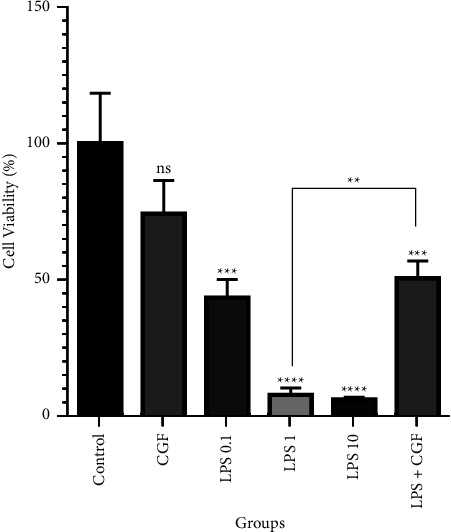
MTT assay for cell viability after 21 days of treatment. Exposure of dental pulp stem cells (DPSCs) to lipopolysaccharide (LPS) combined with concentrated growth factor (CGF) increased the cell viability rates remarkably more than LPS treatment alone; ^*∗∗∗∗*^*p* < 0.0001, ^*∗∗∗*^*p* < 0.001, ^*∗∗*^*p* < 0.01, and ns = nonsignificant.

The cell proliferation rate during treatment of cells with CGF and LPS was investigated using the MTT assay. Exposure to CGF for 21 days exerted a nonsignificant change in the proliferation rate of DPSCs (74 ± 12.24%) compared with the control (100 ± 18.28%). However, treatment of cells with different amounts of LPS inhibited cell proliferation in a dose-dependent way. In comparison with untreated cells, 0.1, 1, and 10 *µ*g/ml of LPS significantly reduced the cell viability to 43 ± 6.65, 7.40 ± 6.65, and 5.78 ± 0.56% (*p* < 0.001, *p* < 0.0001, and *p* < 0.0001), respectively. Besides, when CGF and LPS (1 *µ*g/ml) were simultaneously used, the cell survival rate exhibited a significant (*p* < 0.001) decrease to 50.37 ± 6.32%, which was remarkably higher (*p* < 0.01) than that of LPS 1 *µ*g/ml alone (Figure 4). Tukey's pairwise comparison test showed that the number of DPSCs in the LPS and CGF combination group was significantly higher than in the LPS 1 *µ*g/ml group (*P* < 0.01).

### 3.4. Migratory Ability of Dental Pulp Stem Cells after Long-Term Exposure to Lipopolysaccharide and Concentrated Growth Factor

**Figure 5:**
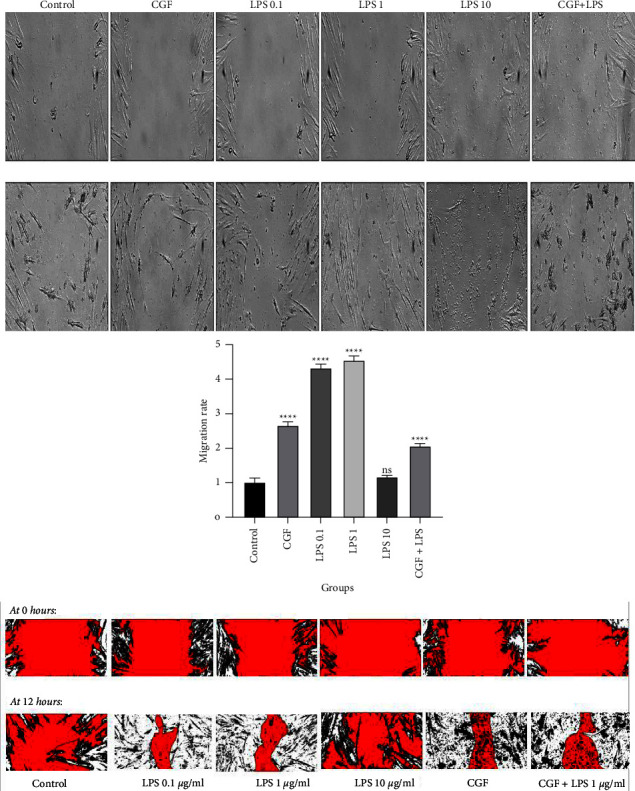
The migration ability of dental pulp stem cells (DPSCs) following treatment according to the scratch assay. The results show that concentrated growth factor (CGF), either alone or in the presence of lipopolysaccharide (LPS), could significantly increase DPSC migration; ^*∗∗∗∗*^*p* < 0.0001 and ns = nonsignificant.

The scratch assay was carried out to explore the combined ability of CGF and LPS to provoke regenerative features in DPSCs in the long term. As shown in Figure 5, although LPS 10 *µ*g/ml had no significant effect on the migratory ability of DPSCs, exposure of these cells to CGF, LPS 0.1 *µ*g/ml, and LPS 1 *µ*g/ml remarkably (*p* < 0.0001) increased the migration rates by 2.65 ± 0.10, 4.32 ± 0.12, and 4.52 ± 0.12 folds compared with the control (1 ± 0.13). Besides, treatment of DPSCs using the combination of CGF and LPS also led to a significant increase (2.068 ± 0.06) in the migration ability of cells. Considering these results, the usage of CGF, separately or in combination with LPS, in long-term conditions could also be considered a promising strategy for the induction of the regenerative feature of migration ability in DPSCs.

### 3.5. Osteogenic Simulation by Concentrated Growth Factor and Lipopolysaccharide

**Figure 6:**
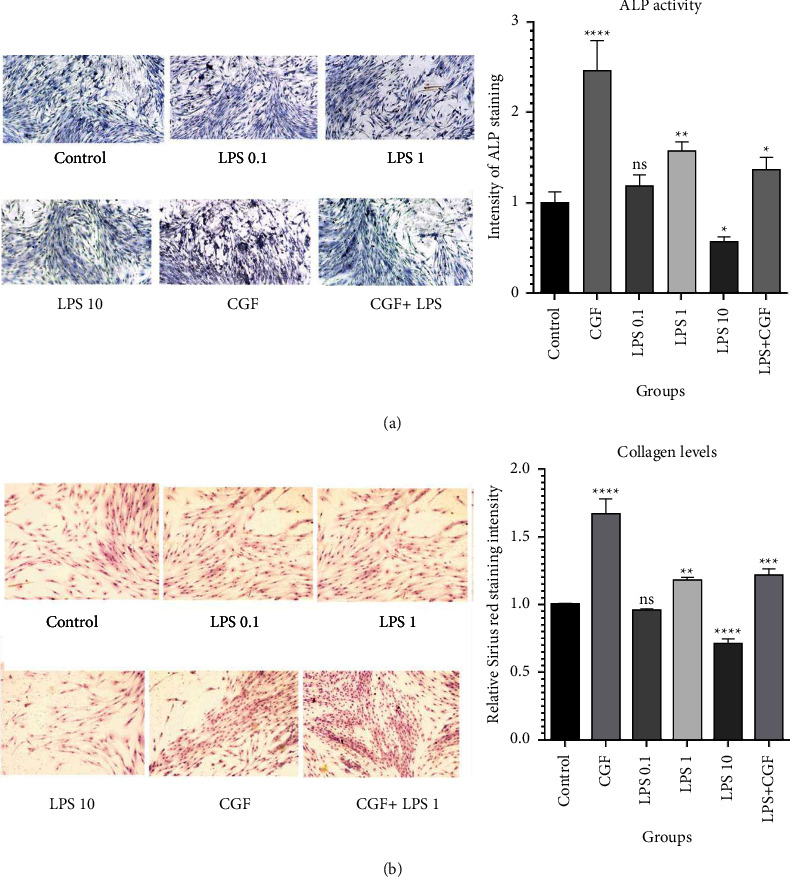
Osteogenic simulation in treatment groups. (a) ALP staining assay; concentrated growth factor (CGF) and lipopolysaccharide (LPS) 1 *µ*g/ml, separately or alone, induced alkaline phosphatase (ALP) activity in dental pulp stem cells (DPSCs). (b) Sirius Red staining assay showing higher levels of collagen in the LPS 1 *µ*g/ml, CGF, and CGF + LPS 1 *µ*g/ml groups; ^*∗∗∗∗*^*p* < 0.0001, ^*∗∗∗*^*p* < 0.001, ^*∗∗*^*p* < 0.01, ^*∗*^*p* < 0.05 and ns = nonsignificant.

The ALP activity assay was carried out to explore the effect of long-term CGF and LPS treatment on the osteogenic properties of DPSCs. As seen in Figure 6(a), although LPS 0.1 *µ*g/ml did not significantly affect ALP activity, CGF and LPS 1 *µ*g/ml each led to a significant (*p* < 0.0001 and *p* < 0.01, respectively) increase in ALP activity by 2.45 ± 0.33 and 1.57 ± 0.10 folds in comparison with the control. In contrast, LPS 10 *µ*g/ml significantly (*p* < 0.05) decreased ALP activity by 0.56 ± 0.05 folds, showing its negative effect on the osteogenic ability of DPSCs. However, CFG combined with LPS also could promote the activity of ALP by 1.36 ± 0.13 folds compared with the control.

In line with this finding, the obtained results from Sirius Red staining (Figure 6(b)) evidenced that treatment of cells with LPS 1 *µ*g/ml could also significantly (*p* < 0.01) increase the collagen levels by 1.18 ± 0.02 folds compared with the control (1 ± 0.007). Also, exposure of cells to CGF alone or combined with LPS 1 *µ*g/ml, respectively, led to a significant (*p* < 0.0001 and *p* < 0.002) increase in collagen production by DPSCs by up to 1.67 ± 0.11 and 1.22 ± 0.04 folds more than the untreated cells. However, LPS 0.1 *µ*g/ml exhibited no significant effect on collagen levels (0.95 ± 0.01 folds), while LPS 10 *µ*g/ml reduced the ability of DPSCs to produce collagen (0.71 ± 0.02 folds, *p* < 0.0001).

**Figure 7:**
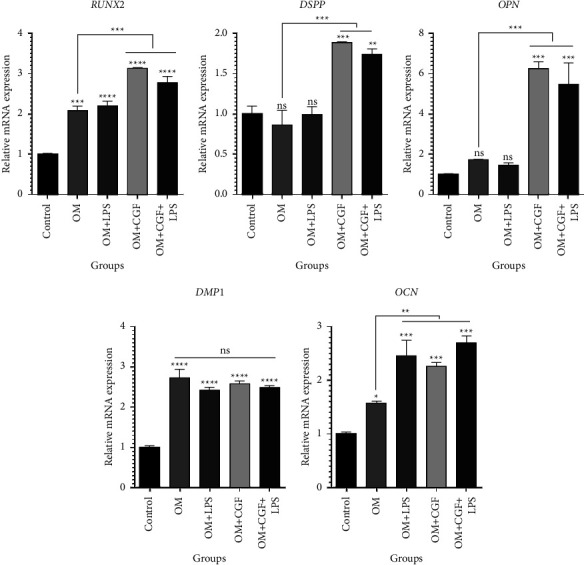
The results of the qRT-PCR assay for the expression levels of osteogenic markers in the treatment groups. ^*∗∗∗∗*^*p* < 0.0001, ^*∗∗∗*^*p* < 0.001, ^*∗∗*^*p* < 0.01, ^*∗*^*p* < 0.05 and ns = non-significant. CGF: concentrated growth factor; LPS: lipopolysaccharide; OM: osteogenic medium.

To further validate osteogenic simulation by treatment groups, qPCR was done to evaluate the expression of osteogenic markers. The results obtained from qPCR illustrated that during long-term exposure, CGF alone or combined with LPS 1 *µ*g/ml could significantly upregulate the expression of RNUX2, DSPP, OPN, and OCN in DPSCs compared to untreated control cells and positive control cells treated with OM alone. However, exposure of cells to isolated LPS only significantly (*p* < 0.001) increased OCN expression (Figure 7). These results suggest that long-term usage of the CGF and LPS combination can promote the osteogenic properties of DPSCs by inducing ALP activity and modulating the expression of osteogenic markers.

## 4. Discussion

Dental pulp regeneration is one of the greatest challenges in dental medicine [18]. Recent studies have shown that inflammation at limited levels serves a great promise to sustain dental pulp vitality and regenerate the dental-pulp complex. However, controlling the range of inflammation in the clinical setting is quite challenging [19, 20]. Some bioscaffolds, such as CGF, may optimize the balance between the inflammatory and regenerative responses in the infected pulp [21]. CGF, as a fibrin scaffold enriched with growth factors, has been used in tissue healing research to promote attachment, proliferation, migration, and differentiation of progenitor cells [21]. CGF may also modulate the balance between LPS-induced inflammatory and regenerative responses in DPSCs [14]. Nevertheless, no study has confirmed this effect in a situation similar to the clinical setting. Therefore, this research intended to investigate the long-term therapeutic effects of CGF on DPSCs with and without LPS-induced inflammation.

The most prevalent causes of inflammation in the dental pulp are tooth decay and microleakage around dental repairs, with microbial infection playing a pivotal role. The response to microbial stimulation leads to inflammation and stimulus persistence induces continuous inflammatory reactions in the pulp that result in pulp necrosis [22]. As common bacteria related to tooth decay, Gram-negative bacteria cause inflammatory reactions via the LPS in the primary membrane [23]. The amount of LPS directly collected from infected root canals can range from 0.001 to 2 *μ*g/ml [14]. Therefore, based on previous studies, three concentrations of LPS (0.1, 1, and 10 *μ*g/mL) were selected to create an effective inflammatory condition stimulating immune responses. In response to LPS, proinflammatory factors and cytokines, such as TNF-*α*, IL-6, and IL-8, are produced and trigger various immune responses in the odontoblasts, fibroblasts, and monocytes of dental pulp tissues [24]. Although short-term treatment with TNF-*α* improved the colony-forming ability, migration, and differentiation of dental pulp cells [25], different durations and doses of treatment may have other effects. Continuous exposure of dental pulp cells to TNF-*α* and IL-1*β* for more than three days reportedly impairs the differentiation ability of cells to odontoblasts, suggesting that chronic secretion of inflammatory cytokines may prevent pulp repair and tissue regeneration [14].

In line with previous reports, the results of this study illustrated that LPS, a potent inducer of TNF-*α*, increased the TNF-*α* expression in DPSCs across the 21-day study period. CGF modulates the expression of proinflammatory cytokines such as TNF-*α* and IL-8 during the LPS-induced inflammatory response in DPSCs, which may accelerate tissue repair [26]. Following these reports, our results indicate that TNF-*α* levels in LPS-induced DPSCs treated with CGF were significantly lower compared with isolated LPS 1 *μ*g/mL treatment. These results suggest that CGF may influence tissue regeneration by controlling and suppressing inflammation in inflamed dental pulp cells.

Our results showed that despite the insignificant effect on cell survival compared with the control, CGF remarkably reduced cell death induced by LPS at 1 *μ*g/mL in DPSCs. These findings indicate that CGF can accelerate the proliferation of DPSCs under LPS-stimulated conditions. CGF can induce the proliferation of several mesenchymal stem cells (e.g., periodontal ligament stem cells (PDLSCs), DPSCs, and hTERT-E6/E7 cells) in a dose-dependent manner [13, 27–29]; though cell proliferation can be inhibited by highly concentrated CGF [30], TGF-*β* and proteolytic enzymes may mediate these effects.

Proinflammatory cytokines secreted by odontoblasts in response to LPS are reportedly responsible for the migration of neutrophils and stem cells [7]. Besides, bFGF and PDGF-BB, as the growth factors of CGF, demonstrated a similar impact on the migration ability of DPSCs compared with granulocyte colony-stimulating factors in vitro. Therefore, CGF treatment may induce the migratory ability in DPSCs and PDLSCs through bFGF and PDGF-BB [31, 32]. The present study indicated that long-term exposure to CGF and LPS (1 *µ*g/ml) could effectively promote the migration ability of DPSCs; the CGF and LPS combination also led to a significant increase in cell migration. Therefore, CGF, by releasing these chemotactic factors, may normalize LPS-induced inflammation and promote the proliferation and migration of DPSCs during long-term chronic inflammation.

An essential step toward pulp regeneration is stem cell differentiation into odontoblasts and the formation of new dentin and capillaries. ALP is an early marker of hard tissue formation or osteoblastic odontoblastic differentiation [33]. Therefore, investigating ALP activity and gene expression related to osteogenic features, such as DMP-1, DSPP, RUNX2, OPN, and OCN, can help to identify the odonto/osteoblastic differentiation of DPSCs. CGF promotes osteogenic/odontoblastic differentiation in DPSCs in vitro by regulating the expression of DMP-1 and DSPP [27]. Also, the inductive effect of CGF on osteogenic differentiation has been confirmed in human bone marrow stem cells [34]. Besides, CGF was reported to increase ALP activity and upregulate the protein expression levels of BMP-2, Col-1, OCN, VEGF, and bFGF in periosteum-derived cells at days 3, 7, 14, and 21 [35]. In the present study, the expression levels of osteo/odontogenic genes increased in the groups containing osteogenic medium (OM) compared with the control group. Our results are supported by a previous study [36] indicating that gingival medicinal signaling cells conditioned medium increased the number of osteoblasts in LPS-induced calvaria bones of Wistar rats. We found that the expression levels of RUNX2, DSPP, and OPN were significantly higher in the CGF + LPS + OM group compared with the LPS + OM group, confirming the controlling effect of CGF on LPS-treated DPSCs. Long-term cotreatment with CGF and LPS significantly promoted ALP activity and collagen production in DPSCs compared with untreated control cells. Further validation of osteogenic simulation by qRT-PCR indicated that the expression level of the DSPP, RUNX2, OPN, and OCN genes remarkably increased in long-term simultaneous treatment with CGF and LPS compared to that with the control. These results suggest that CGF, alone or in the presence of LPS, can induce osteogenic simulation in DPSCs through long-term exposure. According to the results of this study, which demonstrate the ability of CGF to control LPS-induced inflammation and reduce its harm to the regeneration of DPSCs, we hypothesize the clinical effectiveness of CGF in cell-free methods of endodontic regeneration, especially in cases where stem cells are exposed to chronic inflammation caused by pulp disease.

## 5. Conclusion

Building on previous studies, our findings indicate that CGF might have a crucial role in controlling the release of pro-inflammatory cytokines such as TNF-*α*, thereby modulating cell proliferation, migration, and odonto/osteogenic differentiation in DPSCs during long-term exposure to LPS. Given the ability of CGF to modulate the regenerative and osteogenic properties in DPSCs, its therapeutic usage to induce pulp-complex regeneration is an interesting prospect. Further studies are needed to completely understand how CGF could be applied effectively in dental pulp tissue engineering.

## Figures and Tables

**Table 1:** The study groups of the current study.

Group	1	2	3	4	5	6
Description	Control	1 × CGF	0.1 *μ*g/mL LPS	1 *μ*g/mL LPS	10 *μ*g/mL LPS	1 *μ*g/mL LPS and 1 × CGF

CGF: concentrated growth factor and LPS: lipopolysaccharide.

**Table 2:** The primer sequences used in the current study.

No.	Primer name	Sequence (5′–3′)	TM	Per 13 *µ*l (total volume)	Concentration
1	RUNX2-F	GCCTTCAAGGTGGTAG	60	1 *µ*l	4 pmol/L
	RUNX2-R	CGTTACCCGCCATGACAG			
2	H–OSTEOPONTIN-F	GACCTGACATCCAGTAC	60	1 *µ*l	4 pmol/L
	H–OSTEOPONTIN-R	GTTTCAGCACTCTGG TCA			
3	DSPP-F	CCATTCCAGTTCCTCAA	60	1 *µ*l	4 pmol/L
	DSPP-R	TGGCATTTAACTCATCCTGT			
4	BgLAP-F (osteocalcin)	GCAAAGGTGCAGCCTTTG	60	1 *µ*l	4 pmol/L
	BgLAP-R (osteocalcin)	GGCTCCCAGCCATTGATA			
5	BMP-F	TGCGGTCTCCTA AAG	60	1 *µ*l	4 pmol/L
	BMP2-R	AACTCGAACTCGCTCA			
6	H-beta actin-F	CTTCCTTCCTGGGC	60	1 *µ*l	4 pmol/L
	H-beta actin-R	GTCTTTGCGGATGTCC			

## Data Availability

The data used to support the findings of this study are available from the corresponding author upon request.

## Additional Points

*Clinical significance*. The use of CGFs in clinical pulp-complex regeneration is a promising prospect considering its regenerative and osteogenic properties in DPSCs.
